# EGFR/HER2 inhibitor AEE788 increases ER-mediated transcription in HER2/ER-positive breast cancer cells but functions synergistically with endocrine therapy

**DOI:** 10.1038/sj.bjc.6605641

**Published:** 2010-04-13

**Authors:** A H Evans, S Pancholi, I Farmer, A Thornhill, D B Evans, S R Johnston, M Dowsett, L-A Martin

**Affiliations:** 1 Breakthrough Breast Cancer Centre, Institute of Cancer Research, London, SW3 6JJ UK; 2 McElwain Laboratories, Institute of Cancer Research, Sutton, UK; 3 Oncology Research Novartis Institutes for BioMedical Research, Basel, CH-4002 Switzerland; 4 The Royal Marsden Hospital, London, SW3 6JB UK

**Keywords:** breast cancer, aromatase, tamoxifen, letrozole, AEE788, p27

## Abstract

**Background:**

Cross-talk between receptor tyrosine kinases and the oestrogen receptor (ER) is implicated in resistance to endocrine therapy. We investigated whether AEE788 (a combined inhibitor of EGFR, HER2 and VEGFR) plus tamoxifen or letrozole enhanced the individual anti-tumour effects of these agents.

**Methods:**

Breast cancer cell lines modelling endocrine-resistant and -sensitive disease were engineered to express aromatase (A) and examined using proliferation, western blotting and ER-*α* transcription assays.

**Results:**

AEE788 enhanced the anti-proliferative effect of tamoxifen and letrozole in ER^+^ cell lines (MCF-7 2A, ZR75.1 A3 and BT474 A3). This associated with an elevated G1 arrest and nuclear accumulation of p27. It is noteworthy that AEE788 alone or in combination with endocrine therapy increased the expression of progesterone receptor (*PGR*) and *TFF1* in BT474 A3 cells. This may indicate a mechanism of resistance to AEE788 in ER^+^/HER2^+^ breast cancers. In a ZR75.1 A3 xenograft, AEE788 alone or in combination with tamoxifen provided no further benefit compared with letrozole. However, letrozole plus AEE788 produced a significantly greater inhibition of tumour growth compared with letrozole alone.

**Conclusion:**

These data suggest that AEE788 plus letrozole in breast cancer overexpressing HER2 may provide superior anti-tumour activity, compared with single agents.

Over 70% of breast cancers at primary diagnosis express the oestrogen receptor (ER) and require oestrogen (E) for their growth. This has been exploited clinically by the development of endocrine agents such as tamoxifen and aromatase inhibitors (AIs). Recent studies suggest that AIs are superior to tamoxifen in early and advanced breast cancer (Goss *et al*, 2005; Howell *et al*, 2005). Despite advances in the efficacy of AIs, a large proportion of women eventually relapse with endocrine-resistant disease. Clinical studies suggest that expression of HER2 is associated with a decreased response to tamoxifen (Dowsett *et al*, 2001; Gutierrez *et al*, 2005; Arpino *et al*, 2007). Similarly, although neoadjuvant letrozole (AI) is clinically effective in ER^+^/HER2^+^ tumours in the short term, long-term treatment is associated with increased tumour proliferation. This implies that therapeutic resistance to AIs in patients with ER^+^/HER2^+^ breast cancer may manifest later in the clinical course of the disease (Ellis *et al*, 2006).

*In vitro* and *in vivo* models of endocrine-resistant breast cancer allude to a cross-talk between the ER and the receptor tyrosine kinase (RTK) signal transduction pathways. This allows the ER to circumvent the need for steroid hormone because of either ligand-independent activation or downregulation of ER genomic function (Arpino *et al*, 2008; Massarweh *et al*, 2008). Studies suggest that the use of specific receptor tyrosine kinase inhibitors (RTKi) can suppress the proliferation of endocrine-resistant cells (Martin *et al*, 2003; Shou *et al*, 2004; Chu *et al*, 2005) and restrict the onset of resistance (Gee *et al*, 2003, Arpino *et al*, 2007). This provides a strong rationale for the combined use of endocrine agents with RTKi.

AEE788 is a combined inhibitor of EGFR/HER2 and VEGFR tyrosine kinases and has been shown to inhibit the proliferation of EGFR- and HER2-overexpressing cell lines (Traxler *et al*, 2004). On the basis of these data, our aims were (1) to determine whether AEE788, in combination with endocrine therapy, could provide superior therapeutic efficacy both *in vitro* and *in vivo* compared with monotherapy and (2) to identify any significant molecular changes associated with treatment, which may have clinical implications.

As our focus was the inhibitory effect of AEE788 on HER2, we selected a panel of breast cancer cell lines with naturally varying ER and HER2 expression levels that modelled endocrine-resistant and -sensitive disease. These were engineered to express aromatase, allowing the analysis of letrozole, tamoxifen and AEE788 in clinically reflective models.

## Materials and methods

Primary antibodies such as phosphorylated and total ERK1/2, AKT, p27, ER-*α* Ser^118^ and total cyclin D1 were purchased from Cell Signaling Inc, Hitchin, Hertfordshire, UK; total ER (6F11) was from Novacastra Laboratories Ltd, Milton Keynes, Buckinghamshire, UK; and actin (AC-20) was purchased from Sigma, Poole, Dorset, UK; aromatase (MCA2077S) was purchased from AbDSeroTec (Oxford, UK). Secondary antibodies such as anti-mouse and anti-rabbit HRP were obtained from Amersham Pharmacia (Little Chalfont, Nottinghamshire, UK). 17*β*-estradiol (E2) and 4-hydroxytamoxifen (4-OH tamoxifen) were obtained from Sigma. Letrozole and AEE788 were synthesised within the laboratories of Novartis Pharma AG (Basel, Switerland).

### Tissue culture

MCF-7, ZR75.1, BT474 and SKBR3 cell lines expressing aromatase or control backbone (neo) were maintained in phenol red containing RPMI 1640 medium plus 2 mM glutamine, 10 *μ*g ml^−1^ insulin, 10% (v/v) fetal bovine serum (FBS) and 1 mg ml^−1^ G418. For all experiments, cell lines were deprived of steroids for 3 days before seeding by culturing in phenol-red-free RPMI 1640 supplemented with 10% (v/v) dextran-coated charcoal-stripped FBS (DCC-FBS) (Darbre *et al*, 1983).

### Real-time quantitative reverse transcriptase PCR

Gene expression was assessed using a TaqMan ABI 7900 (Applied Biosystems, Warrington, Cheshire, UK) as described previously (Martin *et al*, 2005). The sequences of the primer/probe sets were as follows: PGR: forward 5′-ACCTGAGGCCGGATTCAGAA-3′, reverse, 5′CCACAGGTAAGGACACCATAATGAC-3′, probe, 5′FAM-CCAGAGCCCACAATACAGCTTCGAGTCATT-TAMRA-p-3′ TFF1, forward 5′-GCCCAGACAGAGACGTGTACAG-3′, reverse 5′GTCGAAACAGCAGCCCTTATTT-3′, probe, 5′FAM-CCCCCGTGAAAGACAGAATTGTGGTTT-TAMR-p-3′ ESR1: forward, 5′-TTCTTCAAGAGAAGTATTCAAGGACATAAC-3′, reverse 5′-TCGTATCCCACCTTTCATCATTC-3′, probe, 5′FAM-CCAGCCACCAACCAGTGCACCAT-TAMRA-p-3′ GAPDH was used as the housekeeping gene to normalise the data.

### Cell proliferation assays

Aromatase (A) and control (neo) cell lines were seeded into 12-well plates at c.1 × 10^4^ cells per well for MCF-7, ZR75.1, SKBR3 and 4 × 10^4^ for BT474. Monolayers were treated with a combination of drugs for 6 days. Cell number was determined using a Z1 Coulter Counter (Beckman Coulter, High Wickham, UK). The interaction between AEE788 and 4-OH tamoxifen or letrozole was analysed by the median effect plot method described by Chou and Talalay (1984). Calculation of the combination index (CI) took into account a non-fixed drug ratio and was based on the assumption that the action of the two drugs was mutually non-exclusive for the strict detection of synergism. A CI <1 indicates synergism, CI=1 indicates additive and a CI >1 indicates antagonism.

### Transcription assay

Cell lines were seeded in 24-well plates at 7 × 10^4^ cells per well in DCC medium for all cell lines except BT474, which was seeded at 1 × 10^5^ cells per well. After 24 h, monolayers were transfected by Fugene (Roche, Burgess Hill, West Sussex, UK) with 0.1 *μ*g of EREIItkluc and 0.1 *μ*g of pCH110 overnight, before treatment with the drugs indicated. After treatment for 24 h, luciferase (Promega, Southampton, Hampshire, UK) and *β*-galactosidase (Galacton Star, PE Biosystems, Warrington, Cheshire, UK) activity levels were measured using a luminometer.

### Preparation of whole-cell extracts for immunoblots

Cell monolayers were harvested as described previously (Martin *et al*, 2003), resolved by SDS polyacrylamide gel electrophoresis and transferred to nitrocellulose filters (Schleicher and Schuell, GE Healthcare Maidstone, Kent, UK). Filters were probed with the specific antibodies indicated, as described previously (Martin *et al*, 2003).

### Cell-cycle effects of AEE788, alone or in combination with endocrine agents

Cells were seeded into 10 cm dishes, allowed to acclimatise overnight and then transferred to serum-free medium for 24 h. Monolayers were then treated with drug combinations for 72 h. Cells were fixed and stained with propidium iodide. Cell-cycle analysis was carried out using fluorescence-activated cell sorting (Martin *et al*, 2007).

### Apoptosis assay

Apoptosis was measured with a Cell Death Detection ELISA PLUS kit (Roche) according to the manufacturer's instructions. Cells were seeded into 12-well plates at a density of 1 × 10^5^ cells per well. After 24 h, the cells were transferred to serum-free medium overnight, and thereafter treated for 24 h with drug combinations.

### Human tumour xenografts

Experiments were carried out according to Home Office guidelines and after obtaining approval of the Institute of Cancer Research Ethics Committee. Ovariectomised female Ncr Foxhead nude mice were kept under sterile conditions (six per cage) with free access to food and water. The ZR75.1 A3 cell line was grown as subcutaneous xenografts by passage of 2 mm diameter pieces of tumour. Growth was maintained by intradermal injection of an androstenedione pellet (dose 1.5 mg over 60 days; Innovative Research of America, Sarasota, FL, USA). Once tumours reached a diameter of ∼7 mm, mice were randomised to receive vehicle (10% *N*-methyl-pyrollidone (NMP)/90% polyethylene glycol (PEG300), AEE788 (6.7 mg ml^−1^ in 10% NMP/90% PEG300), tamoxifen (3.3 mg ml^−1^ in 10% NMP/90% PEG300)), letrozole (0.17 mg ml^−1^ in 10% NMP/90% PEG300) or AEE788±tamoxifen or letrozole. All drugs were administered daily by oral gavage for a total of 24 days. Tumour growth was assessed twice weekly by caliper measurements of the two largest diameters. Volumes were then calculated according to the formula *a* × *b*^2^ × *π*/6, where *a* and *b* are orthogonal tumour diameters. Tumour volumes were then expressed as percentage change in volume at the start of treatment (day 0).

### Statistical analysis

Data are presented as ±s.e.m. Differences in the mean of two samples were analysed using Student's unpaired *t*-test, with differences <0.05 being considered significant. For the xenograft study, overall statistical difference was calculated using the Kruskal–Wallis test and statistical differences between individual treatment arms were calculated using the Mann–Whitney test. Repeated measures analysis using multilevel modelling with time as a linear predictor was undertaken in SPSS (SPSS Inc, Woking, Surrey, UK). Ln ((volume at time *t*)/(volume at baseline)) was used as the outcome. Between-mouse variation was treated as a random effect, and linear and quadratic effects for the time effect, including interactions with treatment, were used.

## Results

### Generation and characterisation of Arom-transfected cell lines

**Figure 1:**
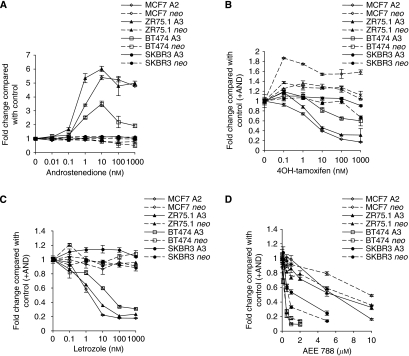
The effect of HER-2 and oestrogen receptor (ER) expressions on the growth response of breast tumour cell lines expressing aromatase to various endocrine agents. (**A**) Breast tumour cell lines with varying ER and HER2 expression levels, transduced to express aromatase (*CYP 19*) (A) or the back bone vector (*neo*), were treated with escalating concentrations of androstenedione. After 6 days of treatment, the cell number was established using a coulter counter. Data are expressed as fold change compared with vehicle control. (**B**) The breast tumour cell lines were treated with log_10_(M) increasing concentrations of 4-OH tamoxifen in the presence of 10 nM androstenedione. (**C**) Cells were treated with log_10_(M) increasing concentrations of letrozole in combination with a standard 10 nM concentration of androstenedione. (**D**) Breast tumour cell lines were treated with increasing concentrations of AEE788 in combination with a standard concentration of androstenedione (10 nM). Bars represent±s.e.m. The data are representative of five individual experiments.

Human breast cancer cell lines with varying endogenous expression levels of ER and HER2 (MCF-7 (ER^+^, HER2^−^), ZR75.1 (ER^+^, HER2^++^), BT474 (ER^+^, HER2^+++^), SKBR3 (ER^−^, HER2^+++^)) were genetically engineered to express aromatase (A) or the backbone vector (*neo)* (Banerjee *et al*, 2009) (Supplementary Figure 1). Treatment of cell lines expressing aromatase with escalating concentrations of androstenedione revealed a concentration-dependent increase in growth for ER^+^ MCF-7 A2, ZR75.1 A3 and BT474 A3, whereas ER^−^ SKBR3 A3 showed no change (Figure 1A). Control clones (*neo*) were non-responsive to the proliferative effects of androstenedione. 4-OH tamoxifen showed a concentration-dependent decrease in growth for MCF-7 A2 (IC50 10 nM) and ZR75.1 A3 (IC50 10 nM) (Figure 1B). BT474 A3 cells were less sensitive with an IC50>1000 nM, whereas SKBR3 A3 cells were unaffected. 4-OH tamoxifen seemed to have a degree of agonist activity in ZR75.1 and BT474 *neo* controls, which was most marked in the MCF-7 *neo* cell line, an observation in keeping with previous studies (Reddel and Sutherland, 1984). Escalating concentrations of letrozole led to a concentration-dependent decrease in proliferation of all ER^+^ cell lines with IC50 values of c.5 nM for ZR75.1 A3 and MCF-7 A2. BT474 A3 cells were less sensitive with an IC50 value of c.50 nM. No effect on SKBR3 A3 was evident (Figure 1C). Letrozole had no effect on the *neo-*expressing control cell lines.

Both BT474 A3 and SKBR3 A3 (Figure 1D) were highly sensitive to the growth-suppressive effects of AEE788 with IC50 values of 0.5 *μ*M and 1 *μ*M, respectively. ZR75.1 and MCF-7 *neo-* and *aromatase-*expressing cells were less sensitive with IC50 values of c.5 *μ*M, indicative of their lower HER2 expression (data shown as colour figure in Supplementary Figure 2).

### Effects of AEE788 alone or in combination with tamoxifen or letrozole on HER2 and ER signalling

**Figure 2:**
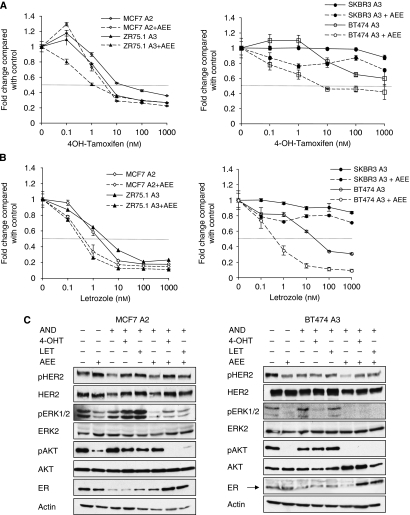
AEE788 functions synergistically with 4-hydroxytamoxifen (4-OH) tamoxifen or letrozole in ER^+^ cell lines. (**A**) ER^+^ cell lines MCF-7 A2 and ZR75.1 A3 were treated with a standard concentration of androstenedione (10 nM) and log_10_(M) increasing concentrations of 4-OH tamoxifen or letrozole±AEE788 (2 *μ*M). After 6 days of treatment, the cell number was analysed using a coulter counter. (**B**) HER2^+^ cell lines BT474 A3 and SKBR3 A3 were treated as described in A, with the exception that 0.25 *μ*M AEE788 was used in the combination. Bars represent ±s.e.m of triplicate wells. The data are representative of three individual experiments. (**C**) The effect of the drugs alone or in combination was assessed by immunoblotting. BT474 A3 and MCF-7 A2 cells were deprived of steroids then treated with a standard concentration of androstenedione (10 nM) alone or in combination with 4-OH tamoxifen (10 nM), letrozole (10 nM) or AEE788 (5 *μ*M for MCF-7 A2, or 0.5 *μ*M for BT474 A3 cells). After 48 h, whole-cell extracts were probed for the markers indicated.

Target cell lines were treated with escalating concentrations of 4-OH tamoxifen or letrozole±a sub-optimal concentration of AEE788 (ZR75.1 A3, MCF-7 A2 (2 *μ*M) and BT474 A3 (0.25 *μ*M)). Combination with AEE788 resulted in a 10-fold increase in sensitivity to 4-OH tamoxifen (IC50 10 nM vs 1 nM) for ZR75.1 A3 and a two-fold increase in sensitivity in MCF-7 A2 (IC50 10 nM vs. 5 nM) (Figure 2A). Formal analysis showed CI <1, indicating a synergistic relationship for AEE788 in combination with 4-OH tamoxifen at concentrations of 1 and 10 nM (CI 0.52 and 0.4, respectively) in ZR75.1 A3 cells and at 10, 100 and 1000 nM (CI 0.26, 0.26 and 0.25, respectively) in MCF-7 2A. In BT474 A3, which showed a reduced sensitivity to 4-OH tamoxifen, combination with AEE788 significantly lowered the IC50 value from >1000 nM to 10 nM with CI <1 at 10, 100 and 1000 nM 4-OH tamoxifen (CI 0.83, 0.85 and 0.84, respectively). As expected, 4-OH tamoxifen alone or in combination with AEE788 had no effect on the proliferation of the ER^−^ cell line SKBR3 A3.

MCF-7 A2 cells showed a five-fold increase in sensitivity to letrozole (IC50 5 nM to 1 nM) when combined with AEE788 with a CI <1 at letrozole concentrations of 0.1 and 1 nM (CI 0.89 and 0.57, respectively) (Figure 2B). Similarly, ZR75.1 A3 showed a 10-fold increase in sensitivity (IC50 5 nM to 0.5 nM) with CI <1 with letrozole concentrations of 1, 10 and 100 nM (CI 0.145, 0.075 and 0.384, respectively). BT474 A3 cells were highly sensitive to the combination, shifting the IC50 value for letrozole to almost 50-fold (IC50 50 nM *vs* 1 nM) with a CI <1 for letrozole concentrations of 1, 10 and 100 nM (CI 0.67, 0.37 and 0.36, respectively).

Assessment of the HER2 downstream signal transduction pathways showed that AEE788±either endocrine agent had little effect on HER2 phosphorylation at Tyr 1248 in either MCF-7 A2 or BT474 A3 (Figure 2C). However, both pERK1/2 and pAKT were suppressed. Whereas ER-*α* protein levels were reduced by the addition of androstenedione in both cell lines, AEE788 in combination with 4-OH tamoxifen or letrozole increased ER-*α* expression.

### Effects of AEE788 in combination with endocrine treatment on cell-cycle progression

**Figure 3:**
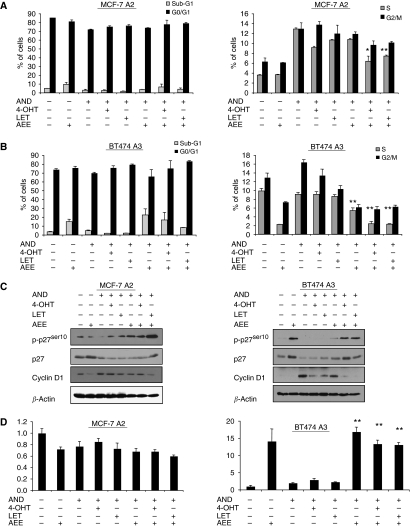
AEE788 in combination with 4-hydroxytamoxifen (4-OH) tamoxifen or letrozole enhances G1 arrest compared with monotherapy. (**A**) Steroid-depleted MCF-7 A2 and (**B**) BT474 A3 cells were treated for 72 h with vehicle, androstenedione (10 nM), 10 nM 4-OH tamoxifen or 10 nM letrozole±AEE788 (5 *μ*M for MCF-7 A2, or 0.5 *μ*M for BT474 A3 cells). Cell cycle was monitored by fluorescence-activated cell sorting analysis of cells stained with propidium iodide (PI). Data are representative of two individual experiments. Bars represent ±s.e.m, ^*^P<0.05, ^**^P<0.01 are derived from the comparison of endocrine agent alone *vs* the combination with AEE788 by Student's unpaired *t-*test (**C**). Duplicate plates treated with drug combinations were harvested after 24 h treatment. Whole-cell extracts were probed for total and phosphorylated p27^Kip1^ and cyclin D1. (**D**) Steroid-depleted MCF-7 A2 and BT474 A3 cells were treated for 24 h with vehicle, androstenedione (10 nM), 10 nM 4-OH tamoxifen or 10 nM letrozole±AEE788 (5 *μ*M for MCF-7 A2, or 0.5 *μ*M for BT474 A3 cells). Apoptosis was measured using a Cell Death Detection ELISA PLUS kit (Roche). Data are expressed as fold increase in apoptosis compared with that in the steroid-treated control. Bars represent ±s.e.m of triplicate wells ^*^P<0.05, ^**^P<0.01 are derived from the comparison of endocrine agent alone *vs* the combination with AEE788 by Student's unpaired *t-*test. Data are representative of two individual experiments.

As both ERK1/2 and AKT are intricately involved in cell growth, we investigated the effect of AEE788±endocrine therapy on cell-cycle progression (Figure 3A). As changes in the percentage of cells in G2/M were only modest, we focused our analysis on S-phase and G1-phase alterations. Androstenedione significantly increased the number of MCF-7 A2 cells in S-phase to 13% compared with the steroid-depleted control (3.6%, *P*=0.0004). Treatment with either 4-OH tamoxifen or letrozole decreased this to 9% (*P*=0.006) and 10% (*P*=0.008), respectively. The combination of AEE788 with 4-OH tamoxifen or letrozole reduced this further compared with monotherapies (6.4 *vs* 9%, *P*=0.05; and 7.44 *vs* 10%, *P*=0.005).

In contrast, BT474 A3 cells showed no significant difference in the number of cells in S-phase in control *vs* androstenedione. Treatment with AEE788±androstenedione significantly reduced the number of cells in S-phase (9.9 *vs* 2.24%, *P*=0.035; and 9.1 *vs* 5.37%, *P*=0.003). 4-OH tamoxifen caused an increase in G1 (70 *vs* 76%, *P*=0.05), whereas letrozole seemed most effective (70 *vs* 79.3%, *P*<0.001). AEE788 in combination with endocrine therapy further reduced the proportion of cells in S-phase compared with endocrine agents alone (AEE788+4-OH tamoxifen 2.4%, *P*<0.001; letrozole+AEE788 2%, *P*<0.001) (Figure 3B). However, there was no significant increase in G1 when comparing 4-OH tamoxifen±AEE788. In contrast, letrozole+AEE788 increased the percentage of cells in G1 (79.3 *vs* 83.4%, *P*=0.005). It is noteworthy that the addition of AEE788 seemed to significantly increase the number of cells in sub-G1, suggesting the fact that it may induce apoptosis particularly in the BT474 A3 cell line.

We next investigated alterations in cyclin D1 and p27^kip1^ (Figure 3C). In BT474 A3 cells, cyclin D1 was suppressed significantly by AEE788±endocrine agents compared with androstenedione alone. MCF-7 A2 cells revealed modest changes in cyclin D1, although AEE788 alone or plus letrozole seemed superior. p27^kip1^ expression in MCF-7 2A cells was unchanged with either 4-OH tamoxifen or letrozole. However, AEE788 alone or combined with letrozole showed a marked increase in p27^kip1^. In BT474 A3 cells, AEE788±letrozole or 4-OH tamoxifen induced greater increases in p27^kip1^ expression than these agents alone.

Phosphorylation of p27^kip1^ is the major regulatory mechanism influencing the protein's abundance. We therefore assessed the level of phosphorylation on p27^kip1Ser10^, which stabilises p27 during G1 arrest (Ishida *et al*, 2000). The MCF-7 2A cells (Figure 3C) showed increased phosphorylation of p27^kip1Ser10^ for all treatments compared with androstenedione, although this was most marked when considering AEE788+letrozole. Assessment of BT474 A3 cells showed a more defined profile in which endocrine agents alone had no effect on p27^kip1Ser10^ phosphorylation, whereas AEE788 in steroid-depleted medium or in combination with 4-OH tamoxifen or letrozole markedly increased its phosphorylation.

On the basis of our previous observation that AEE788 seemed to increase the percentage of cells in sub-G1, we investigated the possibility that AEE788 induced apoptosis (Figure 3D). AEE788 had no effect on apoptosis in MCF-7 2A cells. In contrast, AEE788±endocrine therapy significantly increased apoptosis (*P*<0.001) in the BT474 A3 cell line. These data suggested that the combination of AEE788 with endocrine therapy was most effective in the ER^+^, HER2^+^ cell line BT474 A3, particularly when combined with oestrogen deprivation using letrozole.

### AEE788 enhances ER transcriptional activity

**Figure 4:**
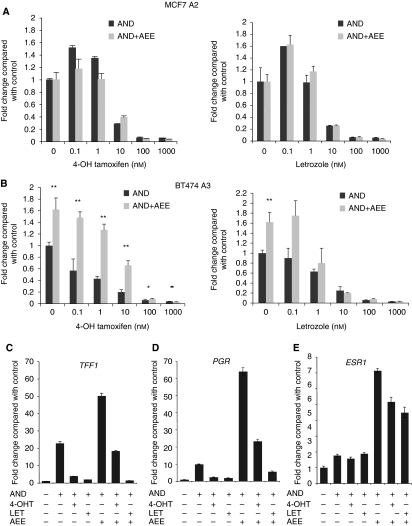
AEE788 enhances oestrogen receptor (ER) transcriptional activity. (**A** and **B**) Cell lines co-transfected with EREIItkLuc and pCH110 were treated with a standard 10 nM concentration of androstenedione and log_10_(M) increasing concentrations of 4-hydroxytamoxifen (4-OH) tamoxifen or letrozole in the absence or presence of AEE788 (5 *μ*M for MCF-7 A2 and 0.5 *μ*M for BT474 A3). Luciferase activity was normalised by *β*-galactosidase from cotransfected pCH110. Normalised luciferase activity from quadruplicate wells was expressed relative to that of the vehicle-treated control. Bars represent ±s.e.m. ^*^P<0.05, ^**^P<0.01, are derived from the comparison of the endocrine agent alone *vs* the combination with AEE788 by Student's unpaired *t-*test. Effects were confirmed in two independent experiments. (**C**) Cells were treated with a standard concentration of androstenedione (10 nM) alone or in combination with 10 nM 4-OH tamoxifen or 10 nM letrozole±0.5 *μ*M AEE788. Quantitative reverse transcriptase PCR was used to measure the expression of *TFF1*, (**D**) progesterone receptor (*PGR*) and (**E**). *ESR1.* Bars represent ±s.e.m.

BT474 A3 and MCF-7 A2 cells were transiently transfected with an ERE-luciferase reporter construct and treated with 4-OH tamoxifen or letrozole±AEE788 (Figures 4A and B). In MCF-7 A2 cells, the combination of drugs provided no further suppression of ER-mediated transactivation compared with endocrine agents alone, and this was confirmed in ZR75.1 A3 cells (data not shown). Low concentrations of 4-OH tamoxifen and letrozole seemed to increase ER-mediated transcription in MCF-7 A2 cells. The reason for this remains unclear. Treatment of BT474 A3 cells with AEE788 alone enhanced ER-mediated transactivation compared with vehicle-treated control (Figure 4B). Increasing concentrations of 4-OH tamoxifen reduced ER-mediated transcription in a concentration-dependent manner but the combination of AEE788±4-OH tamoxifen enhanced ER transcription compared with 4-OH tamoxifen alone at all concentrations tested. Treatment with increasing concentrations of letrozole plus AEE788 suppressed ER-mediated transcription to the same degree as letrozole alone at all concentrations tested.

To gain a broader perspective of the effect of AEE788±4-OH tamoxifen or letrozole on ER-mediated transcription, the expression of two oestrogen-regulated genes, progesterone receptor (*PGR*) and *TFF1*, was measured by quantitative reverse transcriptase PCR in BT474 A3 cells (Figures 4C and D). Androstenedione increased the expression of both target genes compared with the steroid-depleted control. Both 4-OH tamoxifen and letrozole suppressed expression. However, as seen with ER/ERE reporter assays, AEE788 increased the expression of both genes. In addition, AEE788 plus 4-OH tamoxifen showed a greater expression of the two genes compared with 4-OH tamoxifen alone. This enhanced expression was much less pronounced for *PGR* and not observed at all with *TFF1* when AEE788 was combined with letrozole. Further assessment showed that AEE788 alone or in combination with endocrine agents also increased the expression of *ESR1* (Figure 4E) in keeping with our previous observations at the protein level (Figure 2C).

### The effect of AEE788 alone or in combination with letrozole or tamoxifen on the growth of ZR75.1 A3 xenografts

**Figure 5:**
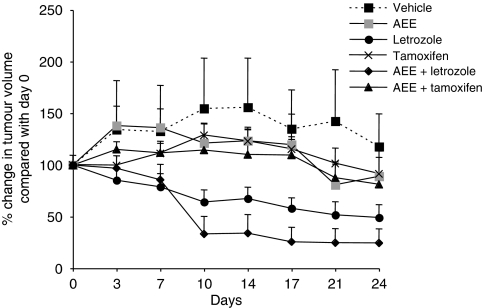
AEE788 in combination with letrozole is more effective at reducing tumour volume compared with either treatment alone. ZR75.1 A3 xenografts were grown in the presence of androstenedione. Once tumours reached the desired size, animals were randomised to receive vehicle, tamoxifen, letrozole, AEE788 or a combination of agents. Tumour volume was measured at the intervals indicated and expressed as percentage change in tumour volume relative to day 0. Bars represent ±s.e.m.

In light of our *in vitro* data and the suggestion of a synergistic interaction between AEE788 and endocrine therapy, we studied the anti-tumour activity of AEE788±tamoxifen or letrozole in mice bearing subcutaneous ZR75.1 A3 breast cancer xenografts. Initial repeated measures analysis indicated that the growth patterns were curved and not compatible with constant growth or shrinkage (Figure 5). In every case, the estimated AEE788+letrozole baseline-corrected tumour size was smaller. Multiple comparison-corrected *P*-values for the 10-day AEE788+letrozole comparison with the other groups were 0.0029 (*vs* vehicle control), 0.004 (AEE788), 0.351 (letrozole), 0.002 (tamoxifen) and 0.007 (AEE788+tamoxifen). Only in the case of the comparison with letrozole did the difference with AEE788+letrozole fail to reach statistical significance, although the trend was for the combination to be more effective. The equivalent *P*-values at 24 days were 0.10, 0.97, 0.99, 0.31 and 0.53, respectively, indicating that, at this later time point, the significant differences seen at 10 days were lost.

## Discussion

On the basis of our increasing knowledge of the interactions between ER and growth factor receptor signalling, there is a rationale for combining RTK inhibitors with endocrine therapy in breast cancer, thus enhancing the efficacy of both agents (Arpino *et al*, 2007). A number of clinical trials investigating this concept have been reported and, although promising, it would seem that only a small proportion of patients show benefit, hence identification of the correct patient population is paramount (Johnston, 2009). The aim of this study was to investigate the predominantly anti-HER2 effects of AEE788 in combination with tamoxifen or letrozole. Our preliminary *in vitro* analysis showed that letrozole was superior at inhibiting the growth of cell lines expressing ER compared with 4-OH tamoxifen, and this was supported by our xenograft study. Of particular note is the fact that letrozole also reduced the growth of HER2-amplified BT474 A3 cells *in vitro*, in keeping with previous clinical observations that HER2^+^ breast cancer seems to be more sensitive to oestrogen deprivation than to tamoxifen (Ellis *et al*, 2001; Dowsett *et al*, 2005).

Submicromolar concentrations of AEE788 induced significant growth inhibition in both BT474 A3 and SKBR3 A3 cells *in vitro*, whereas MCF-7 A2 and ZR75.1 A3 were 20-fold less sensitive in keeping with their relative HER2 expression. AEE788 plus either letrozole or 4-OH tamoxifen showed synergy, providing enhanced growth suppression compared with monotherapies. This was most notable in BT474 A3 cells. We postulate that the increased sensitivity of this cell line to the combination of 4-OH tamoxifen or letrozole with AEE788 is reflective of their increased HER2 expression compared with MCF-7 A2 and ZR75.1 A3.

The lack of any interaction between AEE788 and 4-OH tamoxifen or letrozole in ER-negative SKBR3 A3 cells suggested that the synergy seen in BT474 A3 cells might be explained by their dual expression of HER2 and ER. Previous studies suggest that elevation of pAKT and pERK1/2 as a result of increased HER2 signalling reduces sensitivity to endocrine agents (Arpino *et al*, 2008). This can occur through downregulation of ER, ligand-independent activation, or in the case of resistance to tamoxifen, preferential recruitment of coactivators as opposed to corepressors to tamoxifen-bound ER (Shou *et al*, 2004). It has been demonstrated that inhibition of HER2 signalling with gefitinib in combination with tamoxifen restores corepressor recruitment (Shou *et al*, 2004). These studies allude to the ability of EGFR/HER2 signal transduction pathways to modulate ER phosphorylation and recruitment or assembly of the basal transcription machinery.

The ERE reporter assays showed that the combination of AEE788 with 4-OH tamoxifen or letrozole provided no further suppression of ER-mediated transcription compared with endocrine agents alone in either MCF-7 A2 or ZR75.1 A3 cells. This was not unexpected, as these cell lines are dependent on ER signalling for their proliferation. In contrast, ER/ERE-dependent transcription in BT474 A3 cells treated with AEE788±4-OH tamoxifen was enhanced compared with 4-OH tamoxifen alone. This increase in transactivation, however, was not observed with letrozole. Parallel data were obtained on the expression of two endogenous ER-regulated genes *TFF1* and *PGR*. One potential explanation may be attributed to the relative increase in ER-*α* levels seen in BT474 A3 cells when treated with AEE788 in combination with endocrine therapy. These results indicate that in naturally HER2/ER-expressing tumours, increased oestrogen signalling may occur as a result of inhibiting the growth factor signalling pathway and, conversely, increased growth factor signalling may result as a consequence of inhibiting oestrogen signalling. The exact mechanism remains unclear. However, recent studies have implicated the forkhead box transcription factor FOXO3A, which is capable of mediating ER/ERE transactivation. In a recent study, lapatinib was shown to downregulate AKT, removing the repression of FOXO3A and activating ER transcription (Xia *et al*, 2006). It could be hypothesised that this, coupled with the elevated levels of ER seen in our study, would be sufficient to drive ER-mediated transcription. Hence, although BT474 cells are HER-2 dependent and HER2 RTK inhibitors suppress proliferation, treatment can lead to increased ER-driven transcription and may provide an escape mechanism. This provides yet further rationale for the combined use of RTK inhibitors with letrozole in patients with ER^+^HER2^+^ breast cancer.

The observation that AEE788 in combination with endocrine therapy suppressed proliferation and was associated with decreases in ERK1/2 and AKT led us to investigate the effect on cell-cycle progression. We showed in BT474 A3 cells that AEE788 alone led to a significant sub-G1/G1 arrest and a corresponding decrease in S-phase, which was further enhanced by both 4-OH tamoxifen and letrozole. This observation was similarly seen in MCF-7 A2 cells, although to a lesser degree. It is well established that G1 arrest requires an effective kinase inhibitor protein function (Boehm *et al*, 2002). Hence, we assessed the effect of the drug combinations on cyclin D1 and p27^Kip1^. p27^Kip1^ is necessary for anti-oestrogen-mediated cell-cycle arrest, and studies have shown that enhanced expression of HER2 can lead to the deregulation of p27^Kip1^, leading to anti-oestrogen resistance (Donovan *et al*, 2001; Lenferink *et al*, 2001). In this setting, HER2 activates ERK1/2 and AKT, altering the phosphorylation of p27^Kip1^, thus decreasing its susceptibility to protein degradation (Liang *et al*, 2002; Shin *et al*, 2002). We assessed the phosphorylation status of p27^Kip1^ in MCF-7 A2 and BT474 A3 cells treated with AEE788 alone or in combination. Phosphorylation of p27^Kip1 Ser10^ (which targets p27^Kip1^ for accumulation) in MCF-7 2A cells was increased under all treatment conditions when compared with androstenedione, although this was most notable for the combination of letrozole+AEE788. Similarly, BT474 A3 cells showed high levels of p27^Kip1Ser10^ in response to AEE788 alone or in combination. These alterations in phosphorylation of p27^Kip1Ser10^ largely mirrored the changes in pAKT. Correspondingly, in BT474 A3 cells, cyclin D1, a transcription target of ER (Sabbah *et al*, 1999), was also suppressed by AEE788, alone and in combination, confirming growth inhibition.

Assessment of tamoxifen and letrozole±AEE788 in the ZR75.1 A3 xenograft model showed that letrozole as a monotherapy seemed superior to tamoxifen at inhibiting tumour growth, consistent with recent clinical observations that AIs are superior to tamoxifen (Ellis *et al*, 2001; Thürlimann *et al*, 2005). Rather surprisingly, tamoxifen in combination with AEE788 was also less effective than letrozole alone. Indeed, letrozole as a monotherapy was not significantly less potent than AEE788 combined with letrozole. This may be explained by the fact that ZR75.1 A3 cells use the ER signalling pathway predominantly for growth in this setting.

Overall, these observations suggest that oestrogen deprivation in combination with AEE788, or similar EGFR/HER2 inhibitors, may be superior for the treatment of both *de novo* and acquired ER^+^, HER2^+^ endocrine-resistant breast cancer and may also show potential mechanisms through which resistance to therapy may arise.
