# Supplementary material for: EGFR/HER2 inhibitor AEE788 increases ER-mediated transcription in HER2/ER-positive breast cancer cells but functions synergistically with endocrine therapy
Source: Br J Cancer. 2010 Apr 13;102(8):1235–43. doi: 10.1038/sj.bjc.6605641 (PMC2856013; doi:10.1038/sj.bjc.6605641)
Supplement: Supplementary Figures 1 and 2 [file 6605641x1.ppt]

## Slide 1
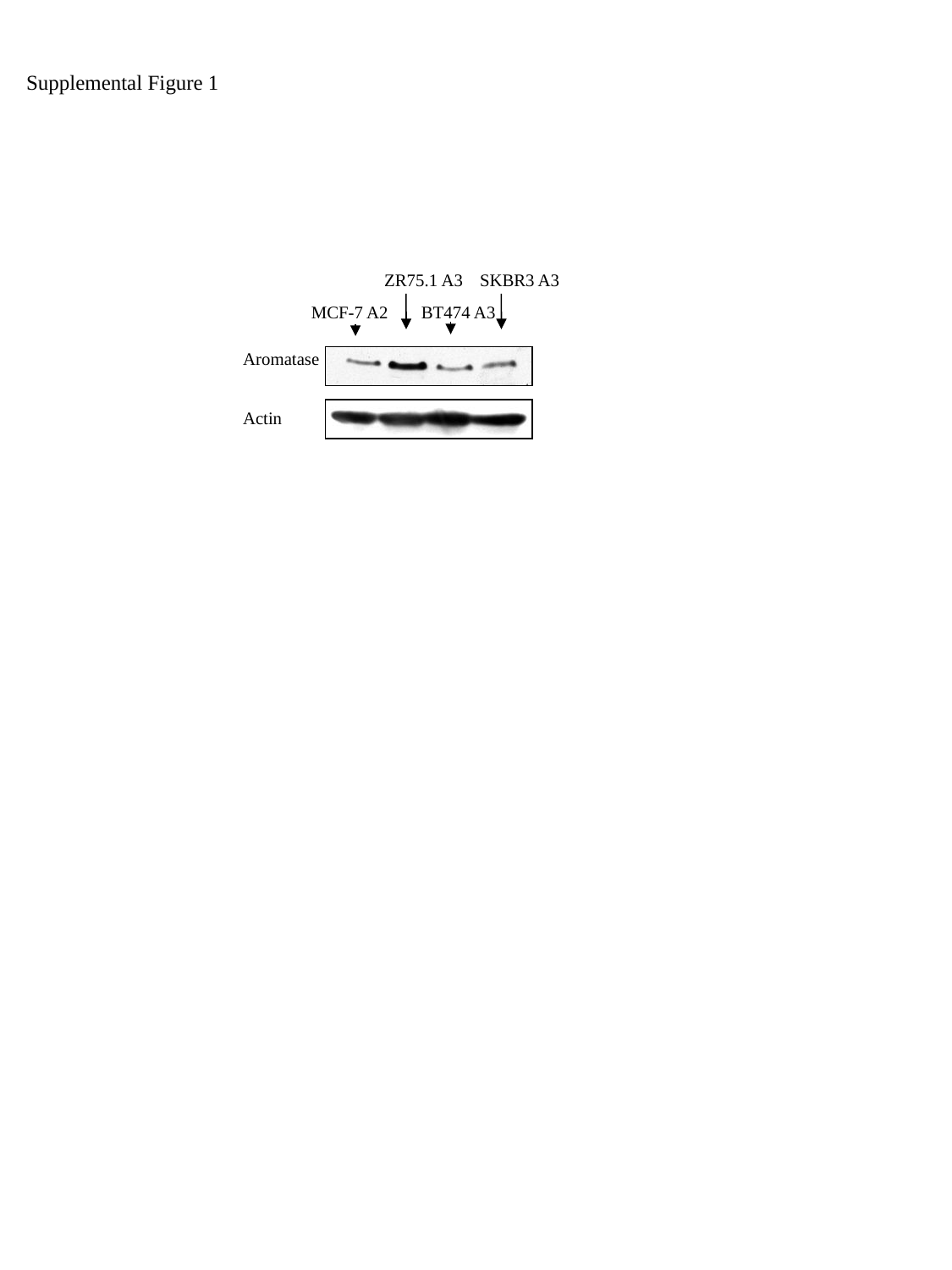

Supplemental Figure 1
ZR75.1 A3
SKBR3 A3
MCF-7 A2
BT474 A3
Aromatase
Actin

## Slide 2
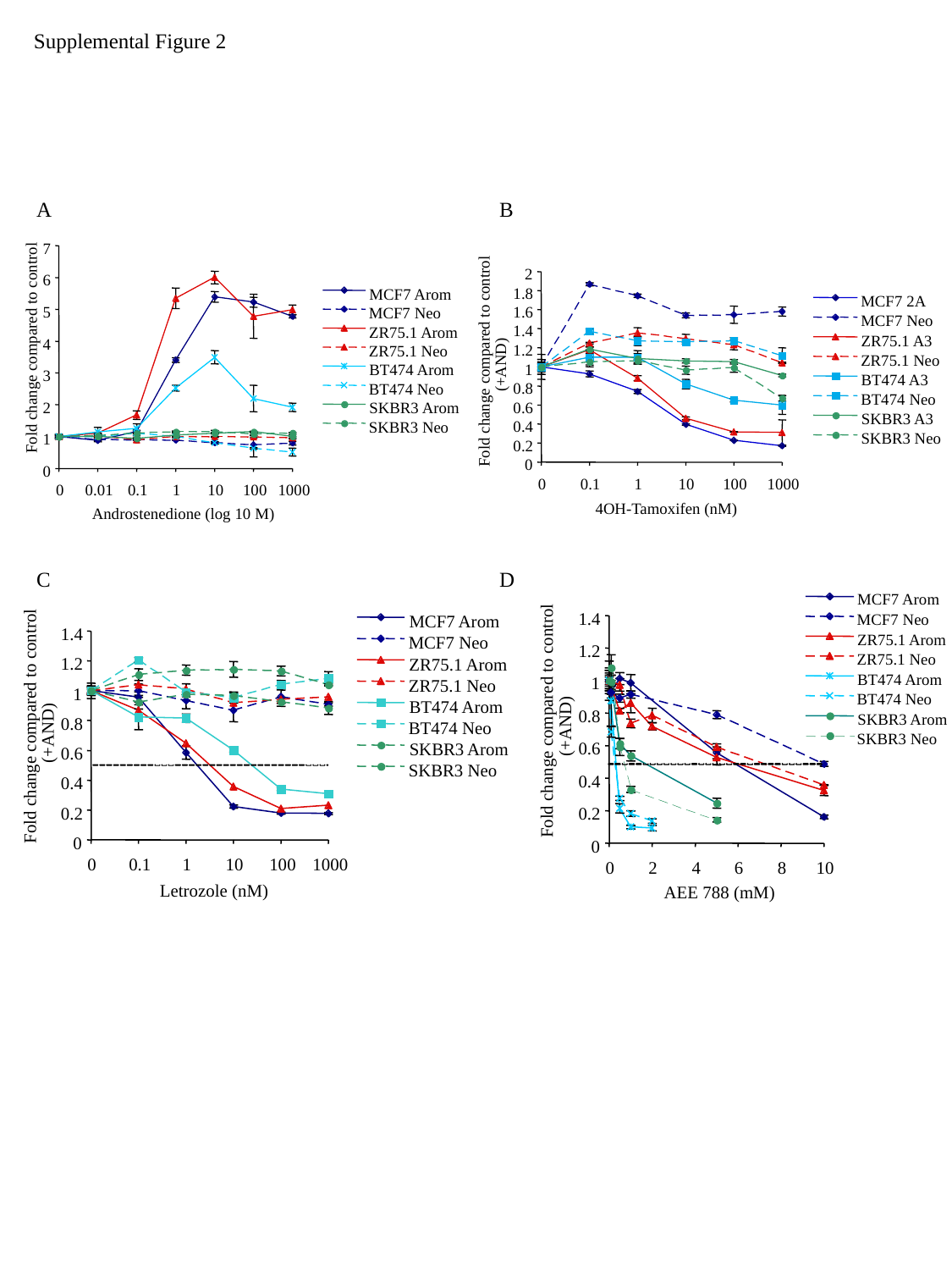

Supplemental Figure 2
A
B
7
6
MCF7 Arom
5
MCF7 Neo
ZR75.1 Arom
4
Fold change compared to control
ZR75.1 Neo
BT474 Arom
3
BT474 Neo
2
SKBR3 Arom
SKBR3 Neo
1
0
0
0.01
0.1
1
10
100
1000
Androstenedione (log 10 M)
2
1.8
1.6
1.4
1.2
1
0.8
0.6
0.4
0.2
0
MCF7 2A
MCF7 Neo
ZR75.1 A3
Fold change compared to control
ZR75.1 Neo
(+AND)
BT474 A3
BT474 Neo
SKBR3 A3
SKBR3 Neo
0
0.1
1
10
100
1000
4OH-Tamoxifen (nM)
MCF7 Arom
1.4
1.2
1
0.8
Fold change compared to control
(+AND)
0.6
0.4
0.2
0
0
2
4
6
8
10
AEE 788 (mM)
MCF7 Neo
ZR75.1 Arom
ZR75.1 Neo
BT474 Arom
BT474 Neo
SKBR3 Arom
SKBR3 Neo
MCF7 Arom
1.4
MCF7 Neo
1.2
ZR75.1 Arom
ZR75.1 Neo
1
BT474 Arom
0.8
Fold change compared to control
BT474 Neo
(+AND)
SKBR3 Arom
0.6
0.4
0.2
0
0
0.1
1
10
100
1000
Letrozole (nM)
SKBR3 Neo
C
D
