# Supplementary material for: EGFR/HER2 inhibitor AEE788 increases ER-mediated transcription in HER2/ER-positive breast cancer cells but functions synergistically with endocrine therapy
Source: Br J Cancer. 2010 Apr 13;102(8):1235–43. doi: 10.1038/sj.bjc.6605641 (PMC2856013; doi:10.1038/sj.bjc.6605641)
Supplement: Supplementary Figures Legends [file 6605641x2.doc]

Supplemental Figures

**Figure 1. Expression of aromatase by *CYP 19* transduced cell lines.**

Whole cell extracts from MCF7 A2, ZR75.1 A3, SKBR3 A3 and BT474 A3 were immunoprobed for expression of aromatase.

**Figure 2**. **The effect of HER-2 and ER expression on the growth response of breast tumour cell lines expressing aromatase to various endocrine agents. Colour version. A.** Breast tumour cell lines with varying ER and HER2 expression levels, transduced to express aromatase (*CYP 19*) (A) or the back bone vector (*neo*) were treated with escalating concentrations of androstenedione. After 6 days of treatment, cell number was established using a coulter counter. Data is expressed as fold change compared to the vehicle control. **B** The breast tumour cell lines were treated with log 10 (M) increasing concentrations of 4-OH tamoxifen in the presence of 10nM androstenedione. **C**. Cells were treated with log 10 (M) increasing concentrations of letrozole in combination with a standard 10nM concentration of androstenedione. **D**. Breast tumour cell lines were treated with increasing concentrations of AEE788 in combination with a standard concentration of androstendione (10 nM). Bars represent ± SEM. The data is representative of 5 individual experiments.
